# Oral anticoagulant prescribing and DOAC dosing appropriateness in patients with non-valvular atrial fibrillation: a retrospective study from Vietnam

**DOI:** 10.1186/s12872-026-06188-8

**Published:** 2026-07-04

**Authors:** Viet Anh Hoang, Tuan Minh Le, Minh Vuong Nong, Van Thuong Bui, Thi Ngoc Anh Tran, Hoai Thi Thu Nguyen, Xuan Co Dao, Van Giap Vu, DaoVu Do

**Affiliations:** 1 Vietnam National Heart Institute, Bach Mai Hospital, Hanoi, Vietnam; 2 Department of Internal Medicine, University of Medicine and Pharmacy, Vietnam National University Hanoi, Hanoi, Vietnam; 3 Institute for Training and Research in Medicine and Pharmacy, Bach Mai Hospital, Hanoi, Vietnam; 4 ICU Center, Bach Mai Hospital, Hanoi, Vietnam; 5 Respiratory Department, Hanoi Medical University, Hanoi, Vietnam; 6 Respiratory Center, Bach Mai Hospital, Hanoi, Vietnam; 7 Rehabilitation Center, Bach Mai Hospital, Hanoi, Vietnam; 8 University of Medicine and Pharmacy, Vietnam National University in Hanoi, Hanoi, Vietnam; 9 Bach Mai Medical College, Hanoi, Vietnam

**Keywords:** Atrial fibrillation, Oral anticoagulants, Direct oral anticoagulants, Vitamin K antagonists, Inappropriate dosing, Prescribing patterns, Real-world evidence

## Abstract

**Background:**

Direct oral anticoagulants (DOACs) are recommended over vitamin K antagonists (VKAs) for stroke prevention in patients with non-valvular atrial fibrillation (NVAF). However, real-world prescribing patterns and dosing appropriateness in low- and middle-income countries remain poorly characterized. This study evaluated patterns of oral anticoagulant use and identified clinical determinants of suboptimal prescribing in a tertiary cardiovascular center in Vietnam.

**Methods:**

We conducted a retrospective observational study of 209 patients with NVAF who received oral anticoagulation at a tertiary cardiovascular center between 2023 and 2024. Data were extracted from electronic medical records, including demographics, comorbidities, renal function, bleeding history, and prescribed anticoagulants. Multivariable logistic regression was used to identify factors associated with anticoagulant selection, defined as DOAC versus VKA use. DOAC dosing appropriateness was assessed according to the 2021 European Heart Rhythm Association Practical Guide, and predictors of potentially inappropriate dose reduction were evaluated.

**Results:**

DOACs were prescribed in 77.0% of patients, most commonly rivaroxaban, which accounted for 55.3% of all anticoagulant prescriptions. VKAs were prescribed in 23.0% of patients. Among VKA users, 85.4% had INR values outside the therapeutic range at admission, largely due to subtherapeutic international normalized ratio values. Reduced DOAC doses were prescribed in 88 of 161 DOAC users (54.7%). Among 85 assessable reduced-dose DOAC users, 42 (49.4%) were classified as having potentially inappropriate dose reduction. In the parsimonious multivariable model, age ≥ 75 years was associated with higher odds of DOAC use (odds ratio [OR] 5.08; 95% confidence interval [CI] 1.95–13.23; *p* = 0.001), whereas female sex (OR 0.43; 95% CI 0.21–0.91; *p* = 0.027), heart failure (OR 0.23; 95% CI 0.09–0.59; *p* = 0.002), and prior bleeding (OR 0.20; 95% CI 0.05–0.87; *p* = 0.031) were associated with lower odds of DOAC use.

**Conclusion:**

In this single-center retrospective study from Vietnam, oral anticoagulant prescribing in patients with NVAF showed several areas for optimization, including frequent INR values outside the therapeutic range at admission among VKA users and frequent potentially inappropriate DOAC dose reduction. These findings support the need for improved dose optimization, and monitoring strategies, but should be interpreted cautiously give the observational design and inpatients data source.

**Supplementary Information:**

The online version contains supplementary material available at https://doi.org/10.1186/s12872-026-06188-8.

## Introduction

Atrial fibrillation (AF) is the most common sustained cardiac arrhythmia and a major contributor to ischemic stroke, morbidity, and mortality worldwide [1–4]. Its prevalence is increasing with population aging, further amplifying the global healthcare burden [5]. Oral anticoagulation is the cornerstone of stroke prevention in patients with non-valvular atrial fibrillation (NVAF) [6–8]. Current guidelines recommend risk stratification using the CHA₂DS₂-VASc score and generally favor direct oral anticoagulants (DOACs) over vitamin K antagonists (VKAs) because of their favorable efficacy and safety profiles [9, 10].

NVAF accounts for most AF cases and represents the primary population for contemporary anticoagulation strategies [11]. VKAs were historically the standard therapy, However, their use is limited by a narrow therapeutic range, multiple food and drug interactions, and the need for regular international normalized ratio (INR) monitoring [12]. DOACs, including dabigatran, rivaroxaban, apixaban, and edoxaban have transformed NVAF management, demonstrating at least comparable efficacy and improved safety, particularly with respect to reduced intracranial bleeding [13, 14]. Accordingly, DOACs are recommended as first–line therapy for most eligible patients with NVAF [11, 15]. Nevertheless, VKAs remain necessary in selected situations, including mechanical heart valves, moderate-to-severe mitral stenosis, severe renal impairment, limited access to DOACs, or cost-related constraints.

Despite clear guideline recommendations, real-world anticoagulant use often deviates from evidence-based practice. Treatment decisions may be influenced by patient age, renal function, comorbidities, bleeding risk, concomitant medications, physician preference, drug availability and affordability [16–20]. In addition, inappropriate DOAC dose reduction is frequently observed in clinical practice and may compromise stroke prevention when dose reduction is not supported by approved criteria or guideline recommendations.

Risk scores such as CHA₂DS₂-VASc and HAS-BLED support clinical decision-making by estimating thromboembolic and bleeding risks, respectively [21–24]. However, these tools provide limited guidance on the selection of a specific anticoagulant agent or on dose optimization in complex patients. As a result, substantial variability in prescribing patterns persists, particularly among older adults and patients with multiple comorbidities, impaired renal function, or previous bleeding events [25–28].

This evidence gap is especially important in low- and middle-income countries (LMICs), where healthcare resources, drug accessibility, medication costs, and monitoring infrastructure may differ substantially from in high-income settings [29]. In Southeast Asia, population-specific characteristics, including lower body weight, higher rates of chronic kidney disease, and differences in healthcare delivery, may further influence anticoagulant selection and dosing [30, 31]. In Vietnam, real-world data remain limited, particularly regarding determinants of anticoagulant choice and the appropriateness of DOAC dosing in patients with NVAF [32].

Therefore, this study aimed to characterize real-world oral anticoagulant prescribing patterns, evaluate the appropriateness of DOAC dosing and identify clinical factors associated with anticoagulant selection among patients with NVAF treated at a tertiary cardiovascular center in Vietnam. By addressing these issues, the study provides context-specific evidence to inform anticoagulation practice and support strategies to improve guideline-concordant stroke prevention in this setting.

## Methods

### Study aim, design, and setting

This study aimed to characterize real-world oral anticoagulant prescribing patterns, assess the appropriateness of DOAC dosing, and identify clinical factors associated with anticoagulant selection among patients with NVAF.

We conducted a retrospective observational study at the Vietnam National Heart Institute (VNHI), Hanoi, Vietnam, a leading tertiary cardiovascular center and a teaching hospital that receives referrals from across the country. This retrospective observational study was reported in accordance with the Strengthening the Reporting of Observational Studies in Epidemiology (STROBE) statement [33]. A study protocol was developed before data extraction and was reviewed and approved by the local Hospital Ethics Committee as part of the ethics approval process. Because this was a retrospective observational study using routinely collected electronic medical record data and did not involve an intervention, the protocol was not prospectively registered in a public trial registry. This retrospective observational study used existing electronic medical record data from patients admitted between 2023 and 2024. Ethical approval for retrospective review of these records was obtained from the local Hospital Ethics Committee on December 15, 2025 (Decision no. 7395/QD-BM). Patient screening, data extraction, and analysis for the present study were performed only after ethics approval had been granted. Because the study involved retrospective analysis of routinely collected, anonymized clinical data and did not involve direct patient contact or intervention, the requirement for informed consent was waived by the Ethics Committee.

### Participants

Eligible participants were adult patients aged ≥ 18 years with a confirmed diagnosis of NVAF who were prescribed oral anticoagulant therapy for stroke prevention during the study period. NVAF was diagnosed based on standard 12-lead electrocardiography or 24-hour Holter monitoring and defined according to the American College of Cardiology/American Heart Association criteria [15].

Patients were included if they had documented NVAF and were receiving either a VKA or a DOAC at the time of admission or during hospitalization. Patients receiving both a VKA and a DOAC during hospitalization were not included in the comparative analysis because switching between anticoagulant classes was not systematically recorded.

Patients were excluded if they had a mechanical heart valve, moderate-to-severe mitral stenosis of rheumatic or non-rheumatic origin or other contraindications to DOAC therapy according to the 2023 ACC/AHA guideline [15]. Pregnant women and patients participating in other study were also excluded.

### Data collection and study variables

After ethics approval was obtained, electronic medical records of inpatients with NVAF admitted during the study period were retrospectively screened according to the predefined inclusion and exclusion criteria. This sampling strategy was used because the inpatient setting allowed systematic data collection from routine clinical care records.

Baseline demographic, clinical, laboratory, and treatment data were extracted from electronic medical records. Demographic variables included age, sex and body mass index (BMI). Age was analyzed both as a continuous variable and as categorical variables using thresholds of ≥ 65 years and ≥ 75 years, corresponding to the age components of the CHA₂DS₂-VASc score [21]. BMI was classified according to the World Health Organization Asia-Pacific criteria as underweight (< 18.5), normal weight (18.5–22.9), overweight (23.0–24.9), and obese (≥ 25.0) [34].

Comorbidities were obtained from medical records and included hypertension, diabetes mellitus, heart failure, prior ischemic stroke or transient ischemic attack, coronary artery disease, chronic kidney disease, prior major bleeding, and anemia. Hypertension was defined as blood pressure ≥ 140/90 mmHg or current antihypertensive treatment [35]; Diabetes mellitus was defined as fasting plasma glucose ≥ 7.0 mmol/L, HbA1c ≥ 6.5%, or use of glucose-lowering therapy [36]; Heart failure was classified according to left ventricular ejection fraction as heart failure with reduced ejection fraction, mildly reduced ejection fraction, or preserved ejection fraction based on European Society of Cardiology criteria [37]. Chronic kidney disease was defined as estimated glomerular filtration rate < 60 mL/min/1.73 m² using the CKD-EPI 2021 equation, in accordance with Kidney Disease: Improving Global Outcomes guidance [38]. Prior major bleeding was defined as Bleeding Academic Research Consortium type ≥ 2 [39]. Anemia was defined according to World Health Organization criteria as hemoglobin < 130 g/L in men and < 120 g/L in women [40].

Laboratory values recorded at the time of DOAC prescription or during the index hospitalization included serum creatinine, hemoglobin, platelet count, and prothrombin time, and international normalized ratio. Cardiac parameters included left ventricular ejection fraction and N-terminal pro-B-type natriuretic peptide. Creatinine clearance was calculated using the Cockcroft–Gault equation because DOAC dose adjustment recommendations are based on creatinine clearance rather than estimated glomerular filtration rate [31].

Thromboembolic risk was assessed using the CHA₂DS₂-VASc score [21]. Patients were categorized as low risk (0 in males or 1 in females), intermediate risk, or high risk (≥ 2 in males, ≥ 3 in females) according to the 2020 European Society of Cardiology guidelines [11]. Bleeding risk was assessed using the HAS-BLED score, with a score ≥ 3 indicating high bleeding risk [41].

Concomitant medications included antiplatelet agents, namely acetylsalicylic acid and clopidogrel; non-steroidal anti-inflammatory drugs, beta-blockers, angiotensin-converting enzyme inhibitors; angiotensin receptor blockers, statins, proton pump inhibitors, digoxin, and amiodarone.

### Anticoagulant exposure and comparisons

Oral anticoagulants were classified as VKAs or DOACs. VKAs included acenocoumarol and warfarin. DOACs included rivaroxaban, apixaban, edoxaban, and dabigatran. Generic drug names were used throughout the study.

The primary comparison was between patients prescribed a DOAC and those prescribed a VKA. Anticoagulant class was analyzed as a binary outcome, coded as 0 for VKA and 1 for DOAC. Among patients receiving DOACs, secondary descriptive analyses evaluated the specific DOAC agent and prescribed dosing regimen.

The explanatory variables were selected a priori based on clinical relevance, guideline-recommended factors for oral anticoagulant selection and dosing, and variables previously reported to be associated with anticoagulant prescribing in patients with NVAF. Variable selection was also constrained by the availability and completeness of data in the electronic medical records. The main explanatory variables assessed as potential determinants of anticoagulant selection included age, sex, comorbidities, thromboembolic risk, bleeding risk, renal function, prior bleeding, and concomitant antiplatelet therapy.

### DOAC dose classification and appropriateness

For patients receiving DOACs, the index DOAC dose used for dose classification and appropriateness assessment was defined as the intended maintenance regimen documented in the discharge prescription at the end of the index hospitalization. If the discharge prescription was unavailable or incomplete, the last active inpatient DOAC order closest to discharge was used, provided that it represented continuation therapy. Temporary inpatient orders, medication holds, peri-procedural interruptions, short-term dose adjustments, and other non-maintenance regimens were not considered the index DOAC dose.

DOAC dose classification and dose-reduction appropriateness were assessed primarily according to the 2021 European Heart Rhythm Association (EHRA) Practical Guide and the approved dose-reduction criteria for each DOAC. These criteria are broadly consistent with product-label dosing recommendations and Vietnamese cardiovascular guidance. Because complete clinical justification for dose reduction was not consistently available in the electronic medical records, reduced-dose prescriptions that did not meet the predefined criteria were classified as “potentially inappropriate” rather than definitively inappropriate.

For rivaroxaban, a dose of 15 mg once daily was considered appropriate in patients with creatinine clearance < 50 mL/min. For apixaban, a dose of 2.5 mg twice daily was considered appropriate when at least two of the following criteria were present: age ≥ 80 years, body weight ≤ 60 kg, or serum creatinine ≥ 133 µmol/L. For dabigatran, a dose of 110 mg twice daily was considered appropriate in patients aged ≥ 80 years or in those with creatinine clearance 30–50 mL/min and high bleeding risk. For edoxaban, a dose of 30 mg once daily was considered appropriate in patients with creatinine clearance 15–50 mL/min, body weight ≤ 60 kg, or concomitant use of potent P-glycoprotein inhibitors. A reduced DOAC dose was considered potentially inappropriate if none of the relevant dose-reduction criteria were met. Full dose-classification criteria are provided in Supplementary Note 1 [31].

### Statistical analysis

All statistical analyses were performed using Python version 3.12 with the statsmodels, SciPy, scikit-learn, and pandas packages. Categorical variables were summarized as counts (n) and percentages (%). Normality of continuous variables was assessed by visual inspection of histograms and Q–Q plots, together with the Shapiro–Wilk test. Because most continuous variables were not normally distributed, they were summarized as medians and interquartile ranges (IQRs).

Missing data were handled using available-case analysis. Patients with missing data required for a specific analysis, such as body weight or laboratory values required for dose-appropriateness assessment, were excluded only from the corresponding denominator. No imputation was performed.

Between-group comparisons were performed according to anticoagulant class, comparing DOAC users with VKA users.The Mann–Whitney U test was used for continuous variables. The chi-square test was used for categorical variables, and Fisher’s exact test was applied when expected cell counts were < 5. Comparisons among individual DOAC agents were performed using the Kruskal–Wallis test for continuous variables and the chi-square test for categorical variables, as appropriate.

Multivariable logistic regression was used to identify factors associated with DOAC prescription after adjustment for covariates included in the model. Variables with *p* < 0.10 in bivariate analysis, together with age and sex, were entered into hierarchical logistic regression models. A parsimonious final model retained only statistically significant predictors. Results were reported as odds ratios (ORs) with 95% confidence intervals (CIs), and statistical significance was assessed using the Wald chi-square test.

To assess the robustness of associations given the limited number of VKA events, six models were evaluated: Model 1 included demographic variables (age ≥ 75, sex); Model 2 added comorbidities; Model 3 added risk factors, Model 4 included all 12 candidate predictors; Model 5 was based on CHA₂DS₂-VASc components, and Model 6 was a parsimonious model including the significant predictors identified in the full model. For events-per-variable calculations, the number of events was conservatively defined as the smaller outcome category, namely VKA use (*n* = 48), in the binary comparison between DOAC and VKA prescription. The full model included 12 candidate predictors, resulting in an events-per-variable ratio of 4.0, whereas the parsimonious model included 4 pre-specified clinical predictors (age, sex, heart failure, and prior bleeding), corresponding to an events-per-variable ratio of 12.0. Five-fold cross-validation was performed to internally assess model performance. Given the limited number of VKA users and the absence of external validation, these multivariable models were considered exploratory association models rather than predictive models. Model discrimination was evaluated using the area under the receiver operating characteristic curve. Calibration was assessed using the Hosmer–Lemeshow test [42]. Internal validation was performed using stratified 5-fold cross-validation. Pairwise area-under-the-curve comparisons were performed using the DeLong test [43] and nested models were compared using likelihood ratio tests [44]. A two-sided p value < 0.05 was considered statistically significant.

## Results

### Patient characteristics and comparison between anticoagulant groups

During the study period, 255 patients with atrial fibrillation receiving oral anticoagulation were screened. Of these, 46 patients were excluded because of incomplete key data required for analysis. Finally, 209 patients with NVAF were included in the final analysis. The median age was 69.0 years (IQR, 64.0–76.0), and 65.1% were male. Persistent or permanent AF was present in 78.9% of patients, paroxysmal AF in 15.8%, and atrial flutter in 5.3%. The distribution of the index arrhythmia did not differ between anticoagulant groups (*p* = 0.42, Fisher’s exact). Median BMI was 22.3 kg/m² (IQR, 19.8–23.9). Heart failure was the most common comorbidity (70.3%), followed by hypertension (53.1%) and anemia (29.7%). The median CHA₂DS₂-VASc score was 3.0 (IQR, 2.0–4.0). Median eGFR was 72.5 mL/min/1.73 m² (IQR, 53.3–89.9), and 68 patients (32.5%) had eGFR < 60 mL/min/1.73 m². Baseline characteristics by anticoagulant class are summarized in Table 1.

**Table 1:** Baseline characteristics of study population by anticoagulant class

Variable	Overall (*n* = 209)	DOAC (*n* = 161)	VKA (*n* = 48)	*p*-value
Age (y), median (IQR)	69.0 (64.0–76.0)	71.0 (63.0–77.0)	68.0 (64.8–72.0)	0.050
Age ≥ 75, n (%)	**66 (31.6)**	**59 (36.6)**	**7 (14.6)**	**0.007**
Male sex, n (%)	136 (65.1)	110 (68.3)	26 (54.2)	0.102
BMI, kg/m², median (IQR)	22.3 (19.8–23.9)	22.6 (20.2–24.2)	21.4 (19.5–23.0)	0.054
Index arrhythmia type, n (%)				
Persistent/permanent AF	165 (78.9)	124 (77.0)	41 (85.4)	0.42†
Paroxysmal AF	33 (15.8)	28 (17.4)	5 (10.4)	
Atrial flutter	11 (5.3)	9 (5.6)	2 (4.2)	
Hypertension, n (%)	111 (53.1)	90 (55.9)	21 (43.8)	0.188
Heart failure, n (%)	**147 (70.3)**	**106 (65.8)**	**41 (85.4)**	**0.015**
Diabetes mellitus, n (%)	48 (23.0)	40 (24.8)	8 (16.7)	0.324
CKD, n (%)	22 (10.5)	15 (9.3)	7 (14.6)	0.438
Prior stroke/TIA, n (%)	36 (17.2)	29 (18.0)	7 (14.6)	0.738
Prior bleeding (BARC ≥ 2), n (%)	10 (4.8)	5 (3.1)	5 (10.4)	0.090
Coronary artery disease, n (%)	36 (17.2)	30 (18.6)	6 (12.5)	0.441
Anemia, n (%)	62 (29.7)	43 (26.7)	19 (39.6)	0.125
eGFR, mL/min/1.73 m², median (IQR)	72.5 (53.3–89.9)	73.3 (56.9–89.0)	63.0 (46.7–92.0)	0.164
CrCl, mL/min, median (IQR)	49.7 (37.5–66.1)	50.6 (38.7–65.7)	43.1 (29.5–68.1)	0.143
Hemoglobin, g/L, median (IQR)	**138 (123–148)**	**138 (126–149)**	**126 (112–141)**	**0.003**
Platelets, ×10⁹/L, median (IQR)	223 (176–274)	224 (179–272)	212 (173–300)	0.775
PT-INR, median (IQR)	**1.2 (1.1–1.7)**	**1.1 (1.1–1.4)**	**1.7 (1.2–3.1)**	**< 0.001**
LVEF, %, median (IQR)	60.0 (41.0–65.0)	60.0 (42.0–65.0)	57.5 (38.5–64.8)	0.399
NT-proBNP, pg/mL, median (IQR)	2170 (964–4970)	2078 (903–4950)	2460 (1192–4906)	0.297
CHA₂DS₂-VASc score, median (IQR)	3.0 (2.0–4.0)	3.0 (2.0–5.0)	3.0 (2.0–4.0)	0.615
Antiplatelet/NSAIDs, n (%)	36 (17.2)	32 (19.9)	4 (8.3)	0.081
Beta-blocker, n (%)	85 (40.7)	63 (39.1)	22 (45.8)	0.508
ACEi/ARB, n (%)	132 (63.2)	105 (65.2)	27 (56.2)	0.337
Statin, n (%)	**111 (53.1)**	**92 (57.1)**	**19 (39.6)**	**0.048**
PPI, n (%)	59 (28.2)	51 (31.7)	8 (16.7)	0.065
Digoxin, n (%)	47 (22.5)	35 (21.7)	12 (25.0)	0.781
Length of stay, days, median (IQR)	7.0 (5.0–10.0)	7.0 (5.0–9.0)	7.0 (5.0–11.0)	0.279

*BMI* Body mass index, *CK*D Chronic kidney disease, *CrCl* Creatinine clearance (Cockcroft-Gault), *eGFR* estimated Glomerular Filtration rate, *PT-INR* Prothrombin time –International normalized ratio, *LVEF* Left Ventricular Ejection Fraction, *NT-proBNP* N-terminal pro–B-type Natriuretic Peptide, *CHA₂DS₂-VASc *C – Congestive heart failure, *H* Hypertension, *A₂* Age ≥75 years (2 points),*D *Diabetes mellitus, *S₂* Prior stroke/transient ischemic attack/thromboembolism (2 points), *V* Vascular disease (coronary artery disease, peripheral artery disease, or aortic plaque), *A* Age 65–74 years, Sc Sex category (female), *NSAIDs *Nonsteroidal Anti-Inflammatory Drugs, *ACEi/ARB* Angiotensin-Converting Enzyme Inhibitors / Angiotensin II Receptor Blockers, *PPI* Proton Pump Inhibitor

† Fisher’s exact test across the three categories (one expected cell <5)

Continuous variables: median (IQR) (Mann-Whitney U test); categorical: n (%) (Chi-square or Fisher’s exact test when expected cell counts <5). Bold p-values indicate statistical significance (p < 0.05)

Oral anticoagulation consisted of DOACs in 161 patients (77.0%) and VKAs in 48 (23.0%). Among VKA users, 41 of 48 patients (85.4%) had INR values outside the therapeutic range at admission. Most were sub-therapeutic, including 28 patients (58.3%) with INR < 2.0 and 20 patients (41.7%) with INR < 1.5) (see Supplementary Table S1). Because these INR values were based on a single measurement at hospital admission, they reflect admission-time anticoagulation status rather than longitudinal VKA control or time in therapeutic range. Among DOAC users, rivaroxaban was most frequently prescribed (55.3%), followed by apixaban (24.8%), dabigatran (9.9%), and edoxaban (9.9%). Among VKA users, acenocoumarol predominated (91.7%), while warfarin was used in 8.3%.

Compared with VKA users, patients receiving DOACs tended to be older median 71.0 (IQR, 63.0–77.0) vs. 68.0 (IQR, 64.8–72.0) years, *p* = 0.050) and were more likely to be aged ≥ 75 years (36.6% vs. 14.6%, *p* = 0.007). Overall clinical characteristics were similar between groups. However, heart failure was more prevalent in the VKA group (85.4% vs. 65.8%, *p* = 0.015). VKA users also had lower hemoglobin levels median 126 (IQR, 112–141) vs. 138 (IQR, 126–149) g/L, *p* = 0.003), while PT-INR was higher as expected median 1.7 (IQR, 1.2–3.1) vs. 1.1 (IQR, 1.1–1.4), *p* < 0.001).

No significant differences were observed in other comorbidities, including hypertension, diabetes, chronic kidney disease, prior stroke, coronary artery disease, or renal function. Thromboembolic risk was comparable between groups (CHA₂DS₂-VASc: median 3.0 (IQR, 2.0–5.0) vs. 3.0 (IQR, 2.0–4.0), *p* = 0.615). Statin use was more frequent in the DOAC group (57.1% vs. 39.6%, *p* = 0.048), whereas other cardiovascular medications and length of hospital stay were similar.

### Comparison among individual DOACs

Baseline characteristics were generally comparable across the four DOAC groups (rivaroxaban, apixaban, edoxaban, dabigatran). No significant differences were observed in age, sex distribution, BMI, renal function (eGFR), left ventricular Ejection Fraction, hemoglobin levels, or most comorbidities, including heart failure and hypertension. Similarly, prior stroke/TIA and prior bleeding did not differ significantly among groups, although prior stroke was numerically more frequent in the apixaban group and prior bleeding in the dabigatran group (Table 2).

**Table 2:** Baseline characteristics among individual DOACs

Variable	Rivaroxaban (*n* = 89)	Apixaban (*n* = 40)	Dabigatran (*n* = 16)	Edoxaban (*n* = 16)	*p*-value
Age, years, median (IQR)	71.0 (63.0–77.0)	72.0 (66.0–81.0)	67.5 (64.5–76.0)	68.0 (63.2–83.5)	0.758
BMI, kg/m², median (IQR)	22.9 (20.9–24.8)	21.5 (19.0–22.9)	23.3 (22.0–24.1)	21.3 (19.8–25.0)	0.066
eGFR, mL/min/1.73 m², median (IQR)	75.0 (59.6–90.3)	65.2 (50.3–83.4)	86.7 (64.2–92.2)	67.6 (57.7–83.1)	0.170
LVEF, %, median (IQR)	59.0 (39.0–65.0)	61.0 (41.8–66.8)	63.5 (57.2–68.0)	60.0 (48.5–63.5)	0.228
Hemoglobin, g/L, median (IQR)	140 (125–154)	139 (130–149)	134 (125–144)	134 (130–146)	0.602
Creatinine, µmol/L, median (IQR)	89.0 (76.0–107.0)	97.0 (78.8–125.8)	77.5 (68.5–101.2)	98.5 (79.5–109.8)	0.119
Male sex, n (%)	64 (71.9)	26 (65.0)	9 (56.2)	11 (68.8)	0.612
Heart failure, n (%)	64 (71.9)	23 (57.5)	9 (56.2)	10 (62.5)	0.330
CKD, n (%)	**6 (6.7)**	**8 (20.0)**	**0 (0.0)**	**1 (6.2)**	**0.048**
Prior stroke/TIA, n (%)	12 (13.5)	12 (30.0)	2 (12.5)	3 (18.8)	0.141
Prior bleeding, n (%)	1 (1.1)	1 (2.5)	2 (12.5)	1 (6.2)	0.093
Hypertension, n (%)	52 (58.4)	20 (50.0)	8 (50.0)	10 (62.5)	0.728
Antiplatelet, n (%)	**25 (28.1)**	**4 (10.0)**	**1 (6.2)**	**2 (12.5)**	**0.035**

Kruskal-Wallis test for continuous variables; Chi-square test for categorical variables. Continuous variables presented as median (IQR); Boldface values indicate statistical significance at *p* < 0.05

Chronic kidney disease differed significantly between groups (*p* = 0.048), with the highest prevalence among apixaban users (20.0%), compared with rivaroxaban (6.7%), edoxaban (6.2%), and none in the dabigatran group. Consistent with this pattern, serum creatinine was higher and mean eGFR lower in the apixaban group, although these differences were not statistically significant. Concomitant antiplatelet therapy also varied significantly (*p* = 0.035), being most common in the rivaroxaban group (28.1%) and less frequent in the apixaban (10.0%), dabigatran (6.2%), and edoxaban (12.5%) groups.

### DOAC dosing patterns and appropriateness

Among 161 DOAC-treated patients, 88 (54.7%) received reduced doses and 72 (44.7%) received standard doses (Table 3). Among 85 assessable patients receiving reduced-dose DOACs, 42 (49.4%) were classified as having potentially inappropriate dose reduction. Patients with missing data required for dose-appropriateness assessment were excluded from the corresponding denominator. Reduced dosing was most frequent with dabigatran (68.8%) and apixaban (67.5%), followed by edoxaban (50.0%) and rivaroxaban (47.2%).

**Table 3:** DOAC dose distribution and appropriateness assessment

DOAC	Standard dose *n* (%)	Reduced dose *n* (%)	Reduced dose appropriate, *n* (%)	Reduced dose potentially inappropriate, *n* (%)
Rivaroxaban (*n* = 89) 20 mg QD/15 mg QD	46 (51.7)	42 (47.2)	22/39 (56.4)	17/39 (43.6)
			CrCl < 50 mL/min*	
Apixaban (*n* = 40) 5 mg BID/2.5 mg BID	13 (32.5)	27 (67.5)	7/27 (25.9)	20/27 (74.1)
			≥ 2 of 3 criteria†	
Dabigatran (*n* = 16) 150 mg BID/110 mg BID	5 (31.2)	11 (68.8)	6/11 (54.5)	5/11 (45.5)
			Age ≥ 80 or CrCl 30–50‡	
Edoxaban (*n* = 16) 60 mg QD/30 mg QD	8 (50.0)	8 (50.0)‡	8/8 (100)	0/8 (0)
			CrCl 15–50 or wt ≤ 60 kg§	
Overall (*n* = 161)	72(44.7)	88 (54.7)	43/85 (50.6)	42/85 (49.4)

* Rivaroxaban: reduced dose 15 mg 15 mg once daily) indicated when CrCl < 50 mL/min. † Apixaban: reduced dose 2.5 mg twice daily indicated when at least two of the following criteria are present: age ≥ 80 years, body weight ≤ 60 kg, serum creatinine ≥ 133 µmol/L (1.5 mg/dL). ‡ Dabigatran: reduced dose 110 mg twice daily indicated when age ≥ 80 years or CrCl 30–50 mL/min with high bleeding risk. §Edoxaban: reduced dose 30 mg once daily indicated when CrCl 15–50 mL/min, body weight ≤ 60 kg, or concomitant P-glycoprotein inhibitors. Patients with missing weight data required for dose-appropriateness assessment (*n* = 3) were excluded from the corresponding denominator. Reduced-dose prescriptions that did not meet the predefined dose-reduction criteria were classified as “potentially inappropriate” because complete clinical justification for dose reduction was not consistently available in the electronic medical records

* DOAC *Direct oral anticoagulant,* QD* Once daily,* BID *Twice daily

Three patients with missing weight data required for dose-appropriateness assessment were excluded from the corresponding denominator. Among 39 assessable patients on reduced-dose rivaroxaban, 22 (56.4%) met the predefined criterion for dose reduction based on CrCl < 50 mL/min. For apixaban, 20 of 27 patients (74.1%) receiving a reduced-dose did not meet ≥ 2 of 3 dose-reduction criteria, and were therefore classified as having potentially inappropriate dose reduction. Dabigatran dose reduction was appropriate in 6 of 11 patients (54.5%) and inappropriate in 5 of 11 patients (45.5%). All 8 edoxaban patients receiving reduced doses met at least one predefined dose-reduction criterion (crCl < 50 or weight ≤ 60 kg). Overall, among 85 assessable reduced-dose patients, 42 (49.4%) were classified as having potentially inappropriate dose reduction (Table 3, see Supplementary Table S4, Figure S3 for details).

### Multivariable analysis of DOAC use in patients with NVAF

**Fig. 1:**
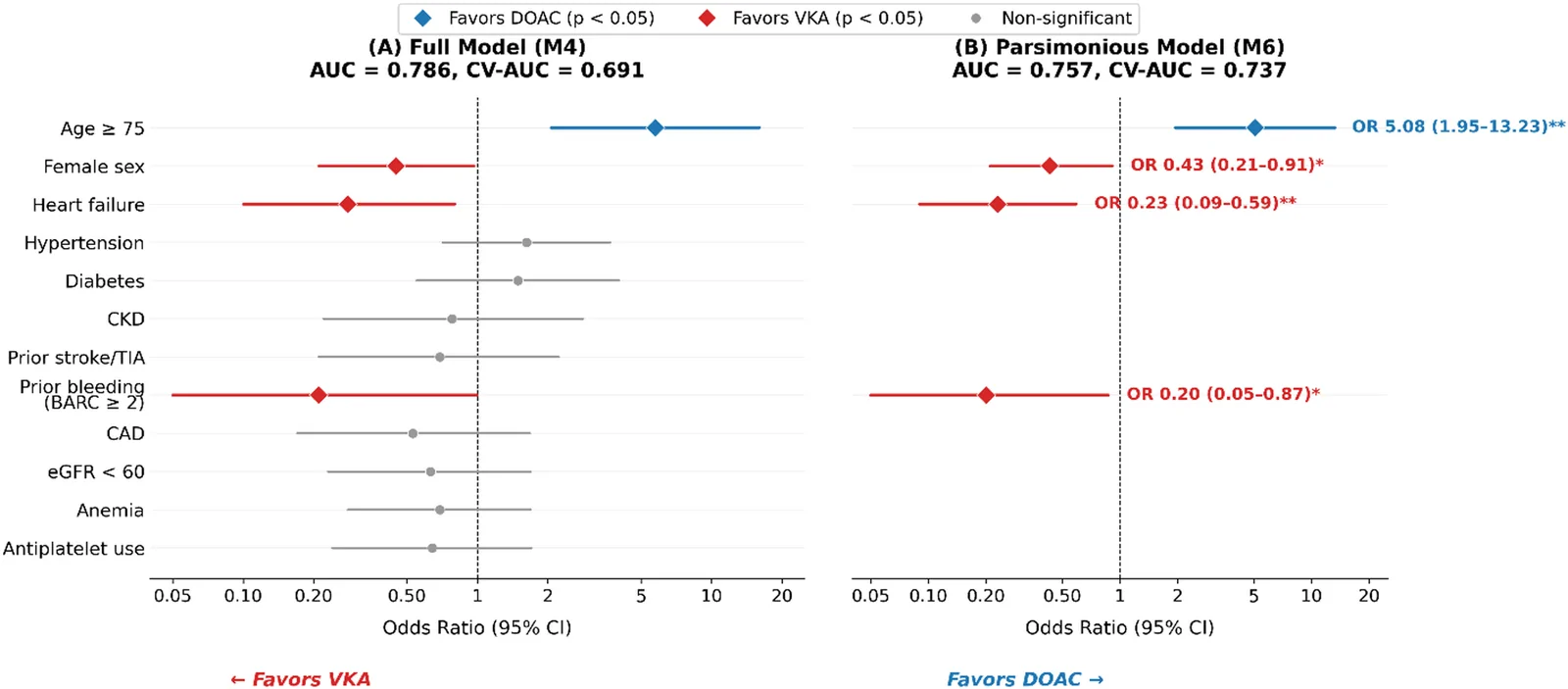
Forest plots of adjusted odds ratios. **A** Full model with 12 predictors (Model 4). **B** Parsimonious model with 4 significant predictors (Model 6). Diamond markers indicate statistically significant variables (*p* < 0.05). OR > 1 favors DOAC; OR < 1 favors VKA. OR: odds ratio; CI: confidence interval. Full model (M4): 12 predictors. Parsimonious model (M6): 4 significant predictors only. Model labels (M4, M6) denote different model specifications tested during model development, Bold p-values: p < 0.05

In the full model (Model 4 - included all candidate predictors), age ≥ 75 years was strongly associated with a higher likelihood of DOAC prescription (OR 5.75, 95% CI 2.07–16.01; *p* < 0.001). In contrast, female sex (OR 0.45, 95% CI 0.21–0.97; *p* = 0.041), heart failure (OR 0.28, 95% CI 0.10–0.80; *p* = 0.017), and prior bleeding (OR 0.21, 95% CI 0.05–1.00; *p* = 0.049) were associated with lower DOAC use. Other clinical factors, including hypertension, diabetes, chronic kidney disease, prior stroke/TIA, coronary artery disease, reduced eGFR, anemia, and antiplatelet use, were not significantly associated with anticoagulant choice (Fig. 1, See Supplementary Table S2 for details).

The parsimonious model (Model 6-a reduced model retaining only selected predictors) showed consistent results. Age ≥ 75 years was associated with higher odds of DOAC prescription after adjustment for covariates included in the model (OR 5.08, 95% CI 1.95–13.23; *p* < 0.001), whereas female sex (OR 0.43, 95% CI 0.21–0.91; *p* = 0.027), heart failure (OR 0.23, 95% CI 0.09–0.59; *p* = 0.002), and prior bleeding (OR 0.20, 95% CI 0.05–0.87; *p* = 0.031) were associated with lower odds of DOAC prescription. Overall, advanced age favored DOAC use, whereas markers of clinical complexity and bleeding risk were associated with preference for VKAs. Other variables (hypertension, diabetes mellitus, CKD, prior stroke/TIA, coronary artery disease, eGFR < 60, anemia, and antiplatelet use) showed weaker or non-significant contributions (Fig. 1, See Supplementary Table S2 for details).

Notably, the full model (M4) had an events-per-variable ratio of 4.0 (48 VKA events for 12 predictors), below the recommended minimum of 10, and showed evidence of overfitting (apparent AUC 0.786 vs. cross-validated AUC 0.691). Therefore, these findings should be interpreted as exploratory associations and should not be considered externally validated predictive results. The parsimonious model (M6), with an events-per-variable ratio of 12, demonstrated more stable performance (apparent AUC 0.757 vs. cross-validated AUC 0.737) and the smallest variability, suggesting better generalizability. It also achieved the lowest AIC (206.6) and BIC (223.3), indicating the optimal balance between model fit and complexity (see Supplementary Figure S1-2 and Table S3). Bootstrap internal validation with 1000 replications showed greater optimism for the full model (M4) than for the parsimonious model (M6). The full model had an optimism of 0.070, an optimism-corrected c-statistic of 0.716, and a calibration slope of 0.64, suggesting substantial overfitting. In contrast, the parsimonious model showed lower optimism (0.020), an optimism-corrected c-statistic of 0.737, and a calibration slope of 0.86, suggesting better internal stability. Nevertheless, because no external validation was performed, these results should be interpreted as exploratory.

## Discussion

DOACs were prescribed in 77.0% of patients, with rivaroxaban most frequently used. This aligns with global trends reported in the GLORIA-AF registry (~ 80% DOAC use) [45] and European data showing utilization exceeding 70% [46], indicating rapid adoption of guideline‑recommended anticoagulation in Vietnam. However, rivaroxaban predominance contrasts with Western practice, where apixaban is most common, suggesting local influences such as drug availability and once‑daily dosing convenience. DOAC use in our study was higher than earlier Southeast Asian reports. The Thai COOL-AF registry described lower early uptake due to cost and access barriers [47], while rivaroxaban predominance reflects regional practice patterns and earlier availability [48]. Clinically, DOAC users were older and more frequently aged ≥ 75 years, consistent with real-world evidence favoring DOACs in elderly patients [39, 49]. In contrast, heart failure and lower hemoglobin were more common in VKA users, suggesting use in more complex patients. Acenocoumarol predominance among VKA users mirrors findings from the Fantas-TIC study [50].

Local prescribing practices may have contributed to OAC selection and dosing patterns in our setting. A recent review by Vietnamese experts noted that NOACs have been used in Vietnam for the past decade and that rivaroxaban is currently the most commonly used NOAC in local clinical practice. The authors also emphasized that anticoagulant selection in Vietnam should consider clinical evidence together with financial factors, insurance coverage, patient preference, adherence, and individual tolerability [51]. These factors may partly explain the predominance of rivaroxaban in our cohort, possibly reflecting local availability, physician familiarity, and the convenience of once-daily dosing. VKA use may persist among patients with cost constraints, limited DOAC access, or clinical complexity requiring closer monitoring. Empirical DOAC dose reduction may reflect a cautious prescribing approach in older or higher-bleeding-risk patients. However, these contextual factors were not systematically captured and should be interpreted as plausible explanations rather than confirmed determinants of OAC selection or dosing.

The proportion of reduced-dose DOAC use (54.7%) in our study was higher than the 20–35% reported in Western registries [49, 52]. According to guideline criteria, dose reduction was appropriate in 56.4% of rivaroxaban, 54.5% of dabigatran and all edoxaban cases, but only 25.9% of apixaban prescriptions; overall, approximately 49% were potentially inappropriate. Similar findings have been reported elsewhere. The CACAO study found 14–26% inappropriate apixaban dose reduction [53], while data from the SAKURA AF registry and Korean cohorts showed that off-label low dosing increased thromboembolic risk without clear bleeding benefit [54, 55]. Observational studies also link potentially inappropriate dose reduction to higher risks of ischemic events, stroke, hospitalization, and mortality [56–58]. These results suggest that empirical dose reduction, often driven by bleeding concerns in elderly or low-weight patients, may compromise thromboembolic protection, highlighting the need for strict adherence to guideline-recommended dosing [48, 53, 55, 56].

Factors associated with DOAC use in patients with NVAF: age ≥ 75 years was the strongest predictor of DOAC prescription (OR 5.08, 95% CI 1.95–13.23, *p* < 0.001). Although some studies and the GLORIA-AF registry reported inconsistent age trends [32, 38], our findings align with European real-world data showing greater DOAC use in older patients to reduce monitoring burden and bleeding risk associated with VKAs [39, 49]. In Vietnam, this may also reflect limited INR monitoring and suboptimal time in therapeutic range (TTR) observed in Asian populations [35]. Heart failure was the strongest negative predictor of DOAC use (OR 0.23, 95% CI 0.09–0.59, *p* = 0.002), despite evidence supporting DOAC safety and efficacy in this population [45, 48]. Clinicians may prefer VKAs due to clinical complexity and reliance on INR monitoring in advanced disease [6, 18, 21]. Female sex was associated in the multivariable modelwith VKA use (OR 0.43, 95% CI 0.21–0.91, *p* = 0.027), contrasting with the Fantas-TIC study where women were more likely to receive DOACs [50]. Sex-related disparities in anticoagulant selection have been inconsistently reported across regions [46, 50], possibly reflecting variations in body weight, comorbidity profiles, or physician perceptions of bleeding risk. Prior bleeding (BARC ≥ 2) was associated in the multivariable model with VKA use (OR 0.20, 95% CI 0.05–0.87, *p* = 0.031), despite strong evidence that DOACs reduce major and intracranial bleeding compared with VKAs [7, 8, 52]. Similar patterns have been reported in European registries [39] and may reflect physician preference for VKAs due to INR monitoring and established reversal strategies [53], particularly where access to DOAC antidotes is limited. This finding highlights a potential evidence–practice gap. Other clinical variables, including hypertension, diabetes, chronic kidney disease, prior stroke/TIA, coronary artery disease, renal dysfunction, anemia, and antiplatelet therapy, were not associated in the multivariable model with anticoagulant selection.

Associations of clinical characteristics and risk scores variables with anticoagulant selection: The CHA₂DS₂-VASc-based model showed limited discrimination for anticoagulant class selection (AUC 0.630; cross-validated AUC 0.601 ± 0.108), and CHA₂DS₂-VASc scores did not differ between DOAC and VKA groups (*p* = 0.615). Although 80.9% of patients were classified as having high stroke risk, the available stroke-risk score variables alone were not sufficient to explain anticoagulant class selection in this dataset. This is consistent with the role of CHA₂DS₂-VASc as a tool for thromboembolic risk stratification and anticoagulation decision-making, rather than for selecting a specific oral anticoagulant agent [5, 10, 12].

In contrast, selected clinical characteristics included in the parsimonious model were associated with DOAC versus VKA prescription. The multivariable clinical model showed better apparent discrimination than the CHA₂DS₂-VASc-based model (AUC 0.786 vs. 0.630; DeLong *p* = 0.001), although its cross-validated performance was lower (cross-validated AUC 0.691). These findings suggest that selected clinical characteristics may help explain anticoagulant class selection in this inpatient dataset. However, because this was a retrospective inpatient study and prescribing decisions and contextual factors were not directly measured, these associations should be interpreted as exploratory rather than causal determinants of prescribing behavior (see Supplementary Figure S1, and Table S3).

These findings suggest potential evidence–practice gaps in anticoagulant management and highlight the need for targeted educational strategies and system-level interventions to optimize DOAC use in resource-constrained settings.

### Study limitations

This study has several limitations. First, the obervational, retrospective, single-center design limits causal inference and may reduce the generalizability of the findings to other healthcare settings.

Second, residual confounding is possible because several potentially important determinants of anticoagulant selection and dosing were not consistently available in the electronic medical records. These included physician preference, patient preference, medication affordability, insurance coverage, socioeconomic status, perceived frailty, adherence, shared decision-making, drug availability, and complete bleeding-risk information. Therefore, the observed associations should be interpreted cautiously as exploratory findings rather than evidence of causal determinants of prescribing behavior.

Third, DOAC dose appropriateness was assessed using data available at prescription or during the index hospitalization. Changes in renal function, body weight, bleeding risk, or concomitant medications during follow-up were not evaluated. In addition, VKA anticoagulation status was assessed using INR values measured at hospital admission. Serial outpatient INR data were not consistently available; therefore, time in therapeutic range could not be calculated. Accordingly, the INR findings should be interpreted as admission-time anticoagulation status rather than long-term VKA control.

Fourth, the multivariable logistic regression analysis was limited by the small number of VKA users. Because only 48 patients received VKAs, the full model including 12 candidate predictors had a low events-per-variable ratio, increasing the risk of overfitting and unstable coefficient estimates. Although a parsimonious model was developed and internally validated using bootstrap resampling and 5-fold cross-validation, these approaches cannot fully overcome the limitation imposed by the low event rate. Because no external validation dataset was available, the multivariable results should be interpreted cautiously and considered exploratory and hypothesis-generating rather than confirmatory or predictive.

Finally, the limited number of patients in individual DOAC subgroups reduced statistical power for agent-specific analyses and may have contributed to imprecise estimates. Despiste these limitations, this study provides real-world evidence on anticoagulant prescribing and DOAC dosing practices in patients with NVAF in a tertiary cardiovascular center in Vietnam.

## Conclusion

In this real-world retrospective cohort of patients with NVAF treated at a tertiary cardiovascular center in Vietnam, DOACs were prescribed more frequently than VKAs, with rivaroxaban being the predominant DOAC and acenocoumarol the most commonly used VKA. Within this inpatient retrospective dataset, selected clinical characteristics were associated with DOAC versus VKA prescription, whereas the role of formal risk scores and unmeasured determinants could not be fully assessed. Frequent potentially inappropriate DOAC dose reduction and VKA use in some higher-risk patients suggest a potential evidence–practice gap. These findings highlight the need for guideline-based prescribing support, regular renal function assessment, dose-optimization, and clinician education to improve anticoagulant management in patients with NVAF.

## Supplementary Information

Supplementary Material 1.

## Data Availability

The datasets generated and/or analyzed during the current study are not publicly available because they contain hospital-based clinical data, but may be available from the corresponding author on reasonable request and with permission from the relevant institutional authorities.

## Abbreviations

ACC: American College of Cardiology
AF: Atrial fibrillation
AHA: American Heart Association
BMI: Body mass index
CHA₂DS₂-VASc: Congestive heart failure, Hypertension, Age ≥ 75 years, Diabetes mellitus, Stroke/transient ischemic attack, Vascular disease, Age 65–74 years, Sex category
CI: Confidence interval
CKD: Chronic kidney disease
CrCl: Creatinine clearance
DOAC: Direct oral anticoagulant
ECG: Electrocardiography
eGFR: Estimated glomerular filtration rate
ESC: European Society of Cardiology
HAS-BLED: Hypertension, Abnormal renal/liver function, Stroke, Bleeding history or predisposition, Labile international normalized ratio, Elderly, Drugs/alcohol
INR: International normalized ratio
IQR: Interquartile range
LMICs: Low- and middle-income countries
LVEF: Left ventricular ejection fraction
NVAF: Non-valvular atrial fibrillation
OR: Odds ratio
PT-INR: Prothrombin time–international normalized ratio
VKA: Vitamin K antagonist
VNHI: Vietnam National Heart Institute
WHO: World Health Organization
