# Supplementary material for: Oral anticoagulant prescribing and DOAC dosing appropriateness in patients with non-valvular atrial fibrillation: a retrospective study from Vietnam
Source: BMC Cardiovasc Disord. 2026 Jul 4;26:838. doi: 10.1186/s12872-026-06188-8 (PMC13613740; doi:10.1186/s12872-026-06188-8)
Supplement: Supplementary file 1 [file 12872_2026_6188_MOESM1_ESM.docx]

**Supplementary documents for**

**Oral anticoagulant prescribing and DOAC dosing appropriateness in patients with non-valvular atrial fibrillation: a retrospective study from Vietnam**

Viet Anh Hoang et al.

# **SUPPLEMENTARY MATERIAL**

**Supplementary Note 1: DOAC dose classification and appropriateness assessment.**

DOAC dose classification and dose-reduction appropriateness were assessed primarily according to the 2021 European Heart Rhythm Association (EHRA) Practical Guide on the use of non-vitamin K antagonist oral anticoagulants in patients with atrial fibrillation. These criteria are broadly consistent with approved product-label dosing recommendations and Vietnamese cardiovascular guidance for DOAC use in patients with non-valvular atrial fibrillation.

For each DOAC, the prescribed dose was classified as either standard or reduced dose based on approved dosing regimens: rivaroxaban 20 mg once daily (standard) or 15 mg once daily (reduced); apixaban 5 mg twice daily (standard) or 2.5 mg twice daily (reduced); dabigatran 150 mg twice daily (standard) or 110 mg twice daily (reduced); and edoxaban 60 mg once daily (standard) or 30 mg once daily (reduced).

Dose-reduction appropriateness was then assessed among patients receiving reduced doses by evaluating whether predefined dose-reduction criteria were met. Renal function for this assessment was estimated using creatinine clearance (CrCl) calculated by the Cockcroft–Gault equation, as recommended by current guidelines and product-label dosing criteria.

The dose-reduction criteria were as follows: rivaroxaban 15 mg once daily if CrCl <50 mL/min; apixaban 2.5 mg twice daily if at least two of the following criteria were present: age ≥80 years, body weight ≤60 kg, or serum creatinine ≥133 µmol/L (1.5 mg/dL); dabigatran 110 mg twice daily if age ≥80 years, or CrCl 30–50 mL/min with concomitant high bleeding risk; and edoxaban 30 mg once daily if CrCl 15–50 mL/min, body weight ≤60 kg, or concomitant use of potent P-glycoprotein inhibitors.

A reduced dose was classified as appropriate if the relevant predefined dose-reduction criteria were fulfilled. Because complete clinical justification for dose reduction, including physician judgment, perceived frailty, patient preference, affordability, adherence concerns, or individualized bleeding concerns, was not consistently available in the electronic medical records, reduced-dose prescriptions that did not meet the predefined criteria were classified as “potentially inappropriate” rather than definitively inappropriate.

.

**SUPPLEMENTARY TABLE LEGENDS**

**Table S1. VKA dose distribution and anticoagulation control.**

| **Variable** | **Acenocoumarol (n=44)** | **Warfarin (n=4)** | **Overall VKA (n=48)** |
| --- | --- | --- | --- |
| **Dose distribution** |  |  |  |
| Daily dose, mg/day, median (IQR) | 1.00 (1.00–1.00) | 1.00 (1.00–1.12) | 1.00 (1.00–1.00) |
| ≤0.5 mg/day, n (%) | 7 (15.9%) | 0 (0.0%) | 7 (14.6%) |
| 0.75–1.0 mg/day, n (%) | 27 (61.4%) | 3 (75.0%) | 30 (62.5%) |
| 1.25–1.5 mg/day, n (%) | 4 (9.1%) | 1 (25.0%) | 5 (10.4%) |
| ≥2.0 mg/day, n (%) | 6 (13.6%) | 0 (0.0%) | 6 (12.5%) |
| INR at admission |  |  |  |
| **INR, median (IQR)** | 1.69 (1.21–2.98) | 2.78 (2.10–4.98) | 1.73 (1.23–3.09) |
| Anticoagulation control |  |  |  |
| **Sub-therapeutic (INR <2.0), n (%)** | 27 (61.4%) | 1 (25.0%) | 28 (58.3%) |
| Therapeutic range (INR 2.0–3.0), n (%) | 6 (13.6%) | 1 (25.0%) | 7 (14.6%) |
| Supra-therapeutic (INR >3.0), n (%) | 11 (25.0%) | 2 (50.0%) | 13 (27.1%) |
| Out of therapeutic range, n (%) | 38 (86.4%) | 3 (75.0%) | 41 (85.4%) |
| Critically sub-therapeutic (<1.5), n (%) | 20 (45.5%) | 0 (0.0%) | 20 (41.7%) |
| Critically supra-therapeutic (>5.0), n (%) | 6 (13.6%) | 1 (25.0%) | 7 (14.6%) |

*INR: international normalized ratio; VKA: vitamin K antagonist. Therapeutic INR range for NVAF: 2.0–3.0. INR values represent a single-point measurement at the time of hospital admission and reflect admission-time anticoagulation status rather than longitudinal VKA control or time in therapeutic range. Acenocoumarol brands: Vincerol, Azenmarol; Warfarin brand: Sintrom.*

**Table S2.** Multivariable logistic regression: predictors of DOAC use (vs VKA).

| **Variable** | **Full Model (M4) OR (95% CI)** | **p** | **Parsimonious (M6) OR (95% CI)** | **p** |
| --- | --- | --- | --- | --- |
| Age ≥ 75 years | 5.75 (2.07–16.01) | **<0.001** | 5.08 (1.95–13.23) | **<0.001** |
| Female sex | 0.45 (0.21–0.97) | **0.041** | 0.43 (0.21–0.91) | **0.027** |
| Heart failure | 0.28 (0.10–0.80) | **0.017** | 0.23 (0.09–0.59) | **0.002** |
| Hypertension | 1.07 (0.49–2.35) | 0.869 | — | — |
| Diabetes mellitus | 1.46 (0.54–3.92) | 0.453 | — | — |
| CKD | 0.58 (0.17–1.92) | 0.369 | — | — |
| Prior stroke/TIA | 0.76 (0.27–2.17) | 0.608 | — | — |
| Prior bleeding (BARC ≥ 2) | 0.21 (0.05–1.00) | **0.049** | 0.20 (0.05–0.87) | **0.031** |
| Coronary artery disease | 0.82 (0.24–2.82) | 0.747 | — | — |
| eGFR < 60 mL/min | 0.71 (0.31–1.64) | 0.427 | — | — |
| Anemia | 0.57 (0.25–1.30) | 0.184 | — | — |
| Antiplatelet use | 2.62 (0.66–10.40) | 0.172 | — | — |

*OR: odds ratio; CI: confidence interval. Full model (M4): 12 predictors. Parsimonious model (M6): 4 significant predictors only. Model labels (M4, M6) denote different model specifications tested during model development,* *Bold p-values: p < 0.05.*

Top of FormBottom of Form

**Table S 3.** Performance comparison of predictive models for DOAC use.

| **Model** | **Variables** | **AUC (apparent)** | **AUC (5-fold CV)** | **Youden's J** | **AIC** | **BIC** | **Brier score** | **HL p-value** |
| --- | --- | --- | --- | --- | --- | --- | --- | --- |
| M1: Demographics | 2 | 0.665 | 0.665 ± 0.075 | 0.228 | 217.6 | 227.7 | 0.1651 | 1.000 |
| M2: Comorbidities | 6 | 0.734 | 0.690 ± 0.067 | 0.396 | 212.1 | 235.5 | 0.1528 | 0.544 |
| M3: Risk Profile | 11 | 0.781 | 0.688 ± 0.122 | 0.519 | 214.2 | 254.3 | 0.1468 | 0.636 |
| M4: Full | 12 | 0.786 | 0.691 ± 0.089 | 0.529 | 214.1 | 257.6 | 0.1450 | 0.876 |
| M5: CHA₂DS₂-VASc | 4 | 0.630 | 0.601 ± 0.108 | 0.252 | 225.3 | 242.1 | 0.1675 | 0.806 |
| M6: Parsimonious | 4 | 0.757 | 0.737 ± 0.070 | 0.409 | 206.6 | 223.3 | 0.1509 | 0.803 |

*AUC: area under the receiver operating characteristic curve; CV: cross-validated; AIC: Akaike information criterion; BIC: Bayesian information criterion; HL: Hosmer–Lemeshow; HF: heart failure; HTN: hypertension; DM: diabetes mellitus; CKD: chronic kidney disease; CAD: coronary artery disease; eGFR: estimated glomerular filtration rate. Values following “±” indicate the standard deviation of the cross-validated AUCs across the 5-fold cross-validation. Shaded row indicates the recommended model. M1: age ≥75 years and sex; M2: M1 + HF, HTN, DM, and CKD; M3: M2 + prior stroke/TIA, prior bleeding, CAD, eGFR <60 mL/min/1.73 m², and anemia; M4: M3 + antiplatelet use; M5: CHA₂DS₂-VASc score, prior bleeding, eGFR <60 mL/min/1.73 m², and anemia; M6: age ≥75 years, sex, HF, and prior bleeding.* .

## **Pairwise model comparisons**

Bootstrap DeLong tests revealed that the parsimonious model (M6) was not significantly inferior to the full model (M4) in discrimination (ΔAUC = −0.029, p = 0.243) or to the risk profile model (M3) (ΔAUC = −0.024, p = 0.227). In contrast, the demographics-only model (M1) was significantly inferior to both M6 (ΔAUC = −0.092, p = 0.001) and M4 (ΔAUC = −0.121, p = 0.002). The CHA₂DS₂-VASc model (M5) was also significantly inferior to M4 (ΔAUC = −0.156, p = 0.001)

Likelihood ratio testing confirmed that adding clinical comorbidities (HF, Hypertension, Diabetes mellitus, CKD) to demographics (M1 → M2) significantly improved model fit (LR χ² = 13.51, df = 4, p = 0.009), but further addition of risk factors (stroke, bleeding, CAD, eGFR<60, anemia) (M2 → M3, LR χ² ≈ 7.9, df = 5, p = 0.158) and medications (antiplatelet) (M3 → M4, LR χ² ≈ 2.04, df = 1, p = 0.153) did not yield significant improvement. At the optimal Youden cutoff (probability = 0.821), the parsimonious model achieved a sensitivity of 59.6% and specificity of 81.2%. **Table** displays the forest plots for both models, and **table** presents model diagnostics. Notably, the full model (M4) had an events-per-variable ratio of 4.0 (48 VKA events for 12 predictors), below the recommended minimum of 10, and showed evidence of overfitting (apparent AUC 0.786 vs. cross-validated AUC 0.691). The parsimonious model (M6), with an events-per-variable ratio of 12, demonstrated more stable performance (apparent AUC 0.757 vs. cross-validated AUC 0.737).

# **Supplementary Table S4. Dose Selection Criteria for DOACs.**

Figure adapted from 2022 Vietnam National Heart Association (VNHA) and Vietnam Heart Rythm Society (VNHRS): DOI: [10.1111/jch.14580](https://doi.org/10.1111/jch.14580?urlappend=%3Futm_source%3Dresearchgate.net%26utm_medium%3Darticle)

# **SUPPLEMENTRY FIGURE LEGENDS**

**Figure S1.** *Model diagnostics. (A) Apparent vs cross-validated AUC across six models. Error bars indicate ±1 standard deviation of the cross-validated AUCs. (B) Calibration plots for the parsimonious (M6) and full (M4) models with Hosmer-Lemeshow p-values. (C) Sensitivity, specificity, and Youden’s J index as a function of probability threshold for M6. (D) Variable importance ranked by Wald χ² statistic for the full model (M4); blue bars indicate p < 0.05, dashed red line indicates χ² = 3.84 (p = 0.05 threshold).*

**Figure S1.** illustrates model diagnostics across four components. (A) Discrimination improved as model complexity increased, with apparent AUC rising from M1 to M4; however, the full model (M4) showed a lower cross-validated AUC, indicating modest overfitting. The parsimonious model (M6) achieved slightly lower apparent discrimination but better and more stable cross-validated performance, suggesting superior generalizability. (B) Calibration plots demonstrated good agreement between predicted and observed probabilities for both M6 and M4, with curves close to the line of perfect calibration and non-significant Hosmer–Lemeshow tests (M6: p = 0.803; M4: p = 0.876). (C) Threshold analysis for M6 showed the expected trade-off between sensitivity and specificity. The optimal cutoff identified by the Youden index was 0.821, corresponding to a sensitivity of 59.6% and specificity of 81.2%, indicating a model that prioritizes specificity. (D) Variable importance in the full model (M4), based on Wald χ² statistics, identified age ≥75 years as the strongest predictor, followed by heart failure, female sex, and prior bleeding (χ² > 3.84, p < 0.05), while other variables contributed minimally. Overall, these findings support the parsimonious model (M6) as providing the best balance between discrimination, calibration, and robustness.

**Figure S2.** *Receiver operating characteristic (ROC) curves for six predictive models of DOAC use vs VKA (n = 209). Dots indicate the optimal Youden cutoff point for each model. AUC: area under the curve; CV: 5-fold cross-validated AUC.*

## **Model development and ROC analysis**

Model performance is summarized in Table 2. Discrimination improved as clinical variables were added, with the full model (M4) achieving the highest apparent AUC (0.786). However, its cross-validated performance declined (CV-AUC 0.691 ± 0.089), indicating modest overfitting.

The parsimonious model (M6), including age ≥75 years, sex, heart failure, and prior bleeding, showed slightly lower apparent discrimination (AUC 0.757) but the best cross-validated performance (CV-AUC 0.737 ± 0.070) and the smallest variability, suggesting better generalizability. It also achieved the lowest AIC (206.6) and BIC (223.3), indicating the optimal balance between model fit and complexity.

Models based solely on limited clinical domains performed less well. The demographics-only model (M1) showed modest discrimination (AUC 0.665), while the comorbidity (M2) and extended risk models (M3) provided incremental but inconsistent improvements with reduced cross-validated performance. The CHA₂DS₂-VASc–based model (M5) had the lowest discrimination (AUC 0.630; CV-AUC 0.601), indicating that stroke risk score alone did not predict anticoagulant selection.

All models demonstrated acceptable calibration (Hosmer–Lemeshow p > 0.05) and similar overall accuracy (Brier score ~0.145–0.168). Overall, the parsimonious model provided the most robust and clinically practical prediction of DOAC use.

# **Supplementary Figure S3. DOAC dosing according to renal function.**

Figure S3. Adapted from 2021 EHRA Practical Guide on the use of DOACs

*Source: Hahn, Kai, and Mathias Lamparter. "Prescription of DOACs in patients with atrial fibrillation at different stages of renal insufficiency." Advances in Therapy 40.10 (2023): 4264-4281.*

^a^110 mg BID in patients at high risk of bleeding (per SmPC). ^b^Other dose reduction criteria may apply (weight ≤ 60 kg, concomitant potent P-Gp inhibitor therapy). According to EMA, SmPC edoxaban should be used in ‘high CrCl only after a careful evaluation of the individual thromboembolic and bleeding risk’. ^c^2 × 2.5 mg only if at least two out of three fulfilled: age ≥ 80 years, body weight ≤ 60 kg, creatinine ≥ 1.5 mg/dl (133 μmol/l). BID, twice daily; CrCl, creatinine clearance; EMA, European Medicines Agency; DOAC, direct oral anticoagulant; RCT, randomised clinical trial; SmPC, Summary of Product Characteristics; VKA, vitamin K antagonist

In this study, reduced-dose prescriptions that did not meet the predefined dose-reduction criteria were described as “potentially inappropriate” because complete clinical justification for dose reduction was not consistently available in the electronic medical records.
